# Childhood Intra-Thoracic Tuberculosis Clinical Presentation Determines Yield of Laboratory Diagnostic Assays

**DOI:** 10.3389/fped.2021.667726

**Published:** 2021-08-25

**Authors:** Urvashi B. Singh, Yogita Verma, Rakhi Jain, Aparna Mukherjee, Hitender Gautam, Rakesh Lodha, Sushil K. Kabra

**Affiliations:** ^1^Department of Microbiology, All India Institute of Medical Sciences, New Delhi, India; ^2^Department of Pediatrics, All India Institute of Medical Sciences, New Delhi, India

**Keywords:** pediatric, intra-thoracic, tuberculosis, primary pulmonary complex, progressive pulmonary disease

## Abstract

Diagnosis of intra-thoracic tuberculosis (ITTB) in children is difficult due to the paucibacillary nature of the disease, the challenge in collecting appropriate specimens, and the low sensitivity of smear microscopy and culture. Culture and Xpert MTB/RIF provide higher diagnostic yield in presumptive TB in adults than in children. Current study was designed to understand poor yield of diagnostic assays in children. Children with presumptive ITTB were subjected to gastric aspirates and induced sputum twice. Samples were tested by Ziehl-Neelsen stain, Xpert MTB/RIF-assay, and MGIT-960 culture. Subjects were grouped as Confirmed, Unconfirmed, and Unlikely TB, and classified as progressive primary disease (PPD, lung parenchymal lesion), and primary pulmonary complex (PPC, hilar lymphadenopathy) on chest X-ray. Of children with culture-positive TB 51/394 (12.9%), culture-negative TB 305 (77.4%), and unlikely TB 38 (9.6%), 9 (2.3%) were smear positive, while 95 (24.1%) were Xpert-MTB/RIF positive. Xpert-MTB/RIF detected 40/51 culture confirmed cases (sensitivity 78.4% and NPV 96.3%). Culture was positive in more children presenting as PPD (*p* < 0.04). In culture-negative TB group, Xpert positivity was seen in 31% of those with PPD and 11.9% in those with PPC (*p* < 0.001).

**Conclusion:** Xpert-MTB/RIF improved diagnosis by 2-fold and increased detection of MDR-TB. Both liquid culture and Xpert-MTB/RIF gave higher yield in children with lung parenchymal lesions. Children with hilar lymphadenopathy without active lung parenchymal lesions had poor diagnostic yield even with sensitive nucleic acid amplification tests, due to paucibacillary/localized disease, suggesting possible utility of invasively collected samples in early diagnosis and treatment.

## Highlights

- Childhood intra-thoracic tuberculosis (ITTB) is difficult to diagnose, despite newer diagnostic tests, with high sensitivity of detection in adults.
- Clinical presentation of ITTB is grouped as progressive primary disease (PPD, lung parenchymal lesion) and primary pulmonary complex (PPC, hilar lymphadenopathy) on chest X-ray.
- Performance of diagnostic tests varies with clinical presentation, being significantly better in children with PPD.
- The yield of diagnostic tests is low with absence of lung parenchymal lesions on x ray chest, suggesting need to use additional methods including endobronchial ultrasound guided aspiration.

## Introduction

Tuberculosis (TB) is one of the leading causes of death among infectious diseases, alongside HIV worldwide. Under-reporting of childhood TB, classification of HIV-TB co-infections as HIV deaths, and underestimated prevalence of pulmonary TB in severely malnourished children are compounded by the spread of MDR and XDR TB strains throughout the world (1).

India, a country with a population of more than 1.3 billion, currently holds a low income, high disease burden country status for TB in children (2). Diagnosing childhood TB is a major challenge due to low sensitivity of clinical criteria for diagnosing TB compounded by poor yield of culture methods and smear microscopy along with the difficulty in obtaining sputum samples, paucibacillary nature of disease in children, and rapid progression from infection to disease (3, 4). Smear microscopy has a sensitivity of 10–15% in pediatric cases, culture-based methods take at least 10–14 days for a positive result, while another 1–2 weeks are lost for drug susceptibility testing by culture-based methods. Intra-thoracic tuberculosis (ITTB) represents the largest burden of disease (75%) in children (5), and sampling methods are most challenging in this group. ITTB in children consists of spectrum of disease manifestations, which can be classified as primary pulmonary complex (PPC) or progressive primary disease (PPD) on the basis of chest X-ray findings (6, 7).

World over, a considerable number of studies are available where the researchers have analyzed the role of Xpert MTB/RIF assay (Cepheid, CA, USA) in diagnosing pulmonary and extrapulmonary tuberculosis, but majority of these have been conducted among adults as the study population (8, 9). The current study was designed to evaluate the role of Xpert MTB/RIF assay in expediting accurate diagnosis of ITTB and MDR-TB in pediatric population and to define factors responsible for poor yield of sensitive diagnostic methods such as Xpert MTB/RIF assay and MGIT 960 culture system in the diagnosis of ITTB in children.

## Methods

A prospective, analytical study was conducted in the Department of Microbiology and Department of Pediatrics, All India Institute of Medical Sciences, AIIMS, New Delhi, from August 2012 to July 2015.

### Study Population and Selection Criteria

Consecutive anti-tubercular therapy (ATT) of naïve children up to 15 years of age, of either sex attending the outpatient department with presumptive ITTB as per guidelines were enrolled in the study (10, 11).

The study population was composed of children with fever and/or cough for 2 weeks with no improvement after 7–10 days course of amoxicillin, recent unexplained weight loss or failure to thrive, unusual/unexplained fatigue, or lethargy, or children with subtle clinical symptoms and history of close contact with suspected or diagnosed case of active TB (12).

### Clinical Data and Treatment

Detailed information regarding the duration of disease, past history of similar illness, general symptoms such as weight loss, cough, appetite, fever, and weakness was obtained from all patients (13). Detailed history, thorough physical examination, Tuberculin Skin Test, radiological findings, hematological, and biochemical findings were recorded in the proforma.

The laboratory was blinded to the clinical and other findings of the patients. The decision to initiate treatment rested with the treating pediatrician, based on clinical definitions (10, 11).

### Sample Collection and Processing

One gastric lavage and one induced sputum were collected from every patient on two consecutive days (13). The study was approved by the Ethics Committee (IEC-IEC/NP-19412012 and RP-ll/2012), All India Institute of Medical Sciences, New Delhi. Consent was taken from the parents/guardians of all the study subjects. Samples were transported to the Mycobacteriology Laboratory and were processed within 4 h of collection. A chest-X-ray and tuberculin skin testing were done for all enrolled children.

Samples for microscopy, culture, and GeneXpert were processed by the NALC-NaOH method i.e., treatment of clinical samples with n-acetyl L-cysteine (NALC), 4% sodium hydroxide, and sodium citrate for 15 min followed by neutralization with PBS (phosphate-buffered saline) and centrifugation at 3,000 rpm for 30 min. The samples were subjected to three tests (14).

Decontaminated samples were stained by Ziehl–Neelsen's technique. Reporting was done as per the National Tuberculosis Elimination Program guidelines (2).

After processing the sample, concentrated sediment was inoculated into MGIT 960 medium and incubated at 37°C. Drug susceptibility testing was performed for positive cultures for first-line drugs by BACTEC™ MGIT™-960 system (as per the instructions from the manufacturer).

The principle of Xpert MTB/RIF assay is based on hemi-nested real-time PCR. The sample was treated as per the instructions from the manufacturer (6, 7).

For the purpose of analysis, the patients were grouped into Confirmed tuberculosis, Unconfirmed, tuberculosis, and Unlikely tuberculosis cases as per international definitions for tuberculosis in pediatric age group (12).

On the basis of chest roentgenography, patients were classified into four groups (i) PPD (progressive primary disease) including parenchymal lesions, (ii) PPC (pulmonary primary complex) presenting as hilar lymphadenopathy, (iii) pleural effusion, and (iv) normal chest X-ray (15, 16).

### Statistical Analysis

Data were analyzed using STATA, ver. 9 (College Station, TX, USA). Categorical variables were described in terms of proportions; continuous variables were described in terms of means and standard deviation (SD). Chi-square test was used to test the significant difference between the proportions. Smear microscopy and Xpert MTB/RIF assay were compared with reference standard (MGIT culture). Measures of diagnostic accuracy (sensitivity, specificity, positive predictive value, and negative predictive value) were calculated.

## Results

A total of 394 outpatient children with presumptive ITTB were enrolled in the study including 206 boys and 188 girls. Seventy-one (18.0%) children were under 5 years of age, 173 (43.9%) children were 5–10 years of age, while 150 (38.1%) were more than 10 years of age.

Based on clinical, microbiological, and radiological criteria, 356 (90.4%) symptomatic children out of the 394 were started on anti-tubercular treatment (ATT). In 38 children, there was no radiological evidence suggestive of TB, symptoms resolved without any ATT, and no new symptoms developed in the follow-up in the next 6 months.

Mean (SD) age of the children initiated on ATT was 8.8 (3.6) years, and 6.9 (3.3) years in the “Unlikely TB” group. The most common symptoms in the treated group were fever (80.1%), loss of appetite (79.5%), weight loss (75.3%), and cough (73.8%). The clinical characteristics of children with presumptive ITTB are outlined in Table 1. Fifty-one (12.9%) and 305 (77.4%) cases belonged to culture-confirmed TB and culture-negative TB groups, respectively. The remaining 38 (9.6%) cases were unlikely TB cases. The children were grouped as PPC and PPD, based on radiological picture. Table 2 summarizes the radiological picture in children in different age groups.

**Table 1:** Clinical, radiological, and immunological profile of the study population.

**Clinical profile**	**Culture-positive TB (*n* = 51)** ***n* (%)**	**Culture-negative TB (*n* = 305)** ***n* (%)**	**No TB (*n* = 38)** ***n* (%)**	***p*-value**
BCG scar present	44 (86.3)	278 (91.1)	33 (86.8)	0.379
Cough	41 (80.3)	222 (72.8)	26 (68.4)	0.403
Fever	47 (92.1)	238 (78)	29 (76.3%)	0.046
Dyspnea	8/21 (38.1)	28/131 (21.3)	1/27 (3.7)	0.01
Night sweats	3/21 (14.3)	6/131 (4.6)	0	0.096
Loss of weight	43 (84.3)	225 (73.8)	23 (60.5)	0.043
Loss of appetite	43 (84.3)	240 (78.7)	29 (76.3)	0.581
History of close contact with a TB case	16 (31.3)	117 (38.4)	7 (18.4)	0.039
Presence of peripheral lymphadenopathy	14 (27.4)	89 (29.2)	8 (21.1)	0.605
Tuberculin Skin Test positivity	47 (92.2)	271 (88.8)	33 (89.2)	0.841
Cough + loss of weight	36 (70.6)	168 (55.1)	15 (39.5)	0.013
Cough + fever + loss of weight	35 (68.6)	147 (48.2)	12 (31.6)	0.002

**Table 2:** Clinical presentation (radiological classification) of intra-thoracic tuberculosis (ITTB) in different age groups.

	**PPD**	**PPC**	**Pleural effusion**	**No TB**
<5 years	24	36	1	10
5–9 years	39	77	10	12
10–14 years	63	91	15	16
Total	126	204	26	38

*P = 0.321.*

*Primary pulmonary complex (PPC): Chest X-ray showing mediastinal adenopathy with or without lung parenchymal lesion.*

*Progressive primary disease (PPD): Chest X-ray showing multiple nodes with parenchymal lesions, consolidation, collapse, cavity, or miliary shadow*.

Smear microscopy, MGIT culture, and Xpert MTB/RIF assay detected (in either of the samples) 2.3% (9/394), 12.9% (51 /394), and 24.1% (95/394) as positive, respectively, a total of 108 (27.4%) cases were microbiologically confirmed. The sensitivity of Xpert MTB/RIF assay (40/51 culture-confirmed TB cases, 78.4%) was higher than that of smear microscopy (6/51 cases, 11.8%) (Tables 3, 4).

**Table 3:** Performance of Xpert MTB/RIF assay and smear microscopy compared with MGIT culture as the reference standard (*n* = 394).

**Diagnostic modality**	**Sensitivity** ** n/N (%)**	**Specificity** ** n/N (%)**	**PPV** ** n/N (%)**	**NPV** ** n/N (%)**
ZN Smear	6/51 (11.8 %)	340/343 (99.1%)	6/9 (66.7%)	340/385 (87.03%)
Xpert MTB/RIF assay	40/51 (78.4%)	288/343 (83.9%)	40/95 (42.1%)	288/299 (95.8%)
**Age group** **<** **5 years**				
ZN smear	1/9 (11.1%)	62/62 (100%)	1/1 (100%)	62/70 (88.6%)
Xpert MTB/RIF assay	8/9 (88.9%)	55/62 (88.7%)	8/15 (53.3%)	55/56 (98.2%)
**Age group 5–9 years**				
ZN smear	1/12 (8.3%)	124/126 (98.4%)	1/3 (33.3%)	124/135 (91.8%)
Xpert MTB/RIF assay	10/12 (83.3%)	109/126 (86.5%)	10/27 (37%)	109/111 (98.2%)
**Age group 10–14 years**				
ZN smear	4/30 (13.3%)	154/155 (99.4%)	4/5 (80%)	154/180 (85.6%)
Xpert MTB/RIF assay	22/30 (73.3%)	124/155 (80%)	22/53 (41.5%)	124/132 (93.9%)
**Hilar lymphadenopathy on chest X-ray**				
ZN smear	3/21 (14.3%)	181/183 (98.9%)	3/5 (60%)	181/199 (91%)
Xpert MTB/RIF assay	14/21 (66.7%)	162/183 (88.5%)	14/35 (40%)	162/169 (95.9%)
**Parenchymal lesion on chest X-ray**				
ZN smear	3/25 (12%)	100/101 (99%)	3/4 (75%)	100/122 (82%)
Xpert MTB/RIF assay	21/25 (84%)	71/101 (70.3%)	21/51 (41.2%)	71/75 (94.7%)
**Pleural effusion on chest X-ray**				
ZN smear	0/2 (0%)	24/24 (100%)	0/0 (0%)	24/26 (92.3%)
Xpert MTB/RIF assay	2/2 (100%)	21/24 (87.5%)	2/5 (40%)	21/21 (100%)
**Induced sputum samples**				
ZN smear	1/51 (2%)	342/343 (99.7%)	1/2 (50%)	343/392 (87.5%)
Xpert MTB/RIF assay	24/51 (47.1%)	308/343 (89.8%)	24/59 (40.7%)	308/335 (91.9%)
**Gastric aspirate samples**				
ZN smear	6/51 (11.8%)	341/343 (99.4%)	6/8 (75%)	341/386 (88.3%)
Xpert MTB/RIF assay	37/51 (72.5%)	299/343 (87.2%)	37/81 (45.7%)	299/313 (95.5%)

**Table 4:** Performance of the diagnostic modalities by categories of TB diagnosis in 394 presumptive pediatric ITTB cases.

**Diagnostic modalities**	**Culture-positive TB** ** (*n* = 51, 12.9%) *n* (%)**	**Culture-negative TB** ** (*n* = 305, 77.4%) *n* (%)**	**Unlikely TB ** **(*n* = 38, 9.6%) *n* (%)**
Smear microscopy positive (*n* = 9)	6 (11.8)	3 (0.98)	0 (0)
MGIT culture positive (*n* = 51)	51 (100.0)	0 (0)	0 (0)
Xpert MTB/RIF positive (*n* = 95)	40 (78.4)	55 (18.03)	0 (0)
**Diagnostic modalities**	**Culture positive and/or Xpert positive TB** ** (** ***n*** **=** **106, 26.9%)** ***n*** **(%)**	**Culture and Xpert negative TB** ** (** ***n*** **=** **250, 63.4%)** ***n*** **(%)**	**Unlikely TB** ** (** ***n*** **=** **38, 9.6%)** ***n*** **(%)**
Smear microscopy positive (*n* = 9)	7 (6.6)	2 (0.8)	0 (0)
MGIT culture positive (*n* = 51)	51 (48.1)	0 (0)	0 (0)
Xpert MTB/RIF positive (*n* = 95)	95 (89.6)	0 (0)	0 (0)

The culture-negative TB group, smear, and GeneXpert assay were positive in 1 and 18%, respectively. Of the 108 microbiologically confirmed cases, MGIT culture was positive/Xpert, MTB/RIF was negative in 11 cases (10.2%), whereas Xpert MTB/Rif was positive when culture was not in 55 cases (50.9%).

Older children (>10 years) had a higher percentage of MGIT culture positivity than the younger children (*p* = 0.02). Children with parenchymal lesion (progressive pulmonary disease, PPD) had a higher percentage of both Xpert MTB/RIF and MGIT culture positivity compared with those with PPC or pleural effusion (*p* < 0.001 and *p* = 0.04, respectively) (Table 5). Among Xpert MTB/RIF positive cases (*n* = 40) in culture-confirmed TB group, 21/40 were PPD, while 14/40 were PPC. In the culture-negative TB group, 30/55 of the Xpert positive cases were PPD, while 21/55 were PPC.

**Table 5:** Age categories and chest X-ray findings in positive MGIT culture and Xpert MTB/RIF assay in children with presumptive TB (*n* = 394).

**Variable**	**Xpert MTB/RIF**		***p*-value**	**MGIT culture**		***p*-value**
	**Positive (*n* = 95)**	**Negative (*n* = 299)**		**Positive (*n* = 51)**	**Negative (*n* = 343)**	
**Age groups**
<5 years (*n* = 71)	15 (15.8)	56 (18.7)	0.14	9 (17.6)	62 (18.1)	0.14
5–9 years (*n* = 138)	27 (28.4)	111 (37.1)		12 (23.5)	126 (36.7)	
>10 years (*n* = 185)	53 (55.8)	132 (44.1)		30 (58.8)	155 (45.2)	
**Chest X-ray findings**
Parenchymal lesion (*n* = 126)	51 (53.7)	75 (25.1)	<0.001	25 (49.0)	101 (29.4)	0.04
Hilar lymphadenopathy (*n* = 204)	35 (36.8)	169 (56.5)		21 (41.2)	183 (53.3)	
Pleural effusion (*n* = 26)	5 (5.3)	21 (7.0)		2 (3.9)	24 (7.0)	
No abnormalities (*n* = 38)	4 (4.2)	34 (11.4)		3 (5.9)	35 (10.2)	

*Definitions as per WHO (13)*.

Test performance for different age groups, chest X-ray findings, and type of samples are given in Table 3.

No statistically significant association was found between the Xpert MTB/RIF assay and MGIT culture positivity with a positive history of close contact with TB case, TST positivity, or presence of BCG scar (data not shown).

In culture-confirmed TB group, smear microscopy gave a higher detection in gastric aspirate (11.8%) compared with induced sputum (1.96%) (*p* 0.0253). Culture detection was higher in the gastric aspirate (72.5%) than that in the induced sputum (50.9%) samples. Similarly, Xpert MTB/RIF assay detection was higher in gastric aspirate (72.5%) compared with induced sputum (47.05%).

Of the 356 patients who were initiated on ATT, gastric aspirate and induced sputum were available for 333 patients, while for the rest, only the gastric aspirate result was available. Induced sputum detected four cases, which were missed on gastric aspirate in culture and five in Xpert MTB/RIF assay. Forty-two gastric aspirate samples were MGIT culture positive with negative corresponding induced sputum samples. Similarly, in Xpert MTB/RIF assay result, an additional 42 positives were detected in gastric aspirate samples compared with induced sputum.

## Discussion

The present study was done exclusively in children (<15 years of age). A total of 394 consecutive outpatients of presumptive intra-thoracic tuberculosis were enrolled. Fever, cough of more than 2-week duration, loss of weight, and loss of appetite among others, are the most common symptoms in TB patients as reported in published literature (4, 5).

Culture is the currently used reference diagnostic method for the diagnosis of ITTB in children. This method suffers from a drawback that it has a poor sensitivity (<30–40%). Culture yield in the pediatric population is highly variable due to various reasons like inadequate sampling, type, and number of samples used for diagnosis, prior antibiotic therapy, and quality of sample (adequately representative of pathology). In the current study, enrolled cases were defined as Confirmed TB, Unconfirmed TB, and Unlikely TB. Culture positive cases in the present study were 12.9% (51/394) of the study population, which is in concordance with studies published from different parts of world with positivity ranging from 6.2 to 17.1% (17–21). This group consisted of 50.25% children with PPD.

Smear microscopy detected nearly one-eighth of the culture-confirmed TB cases, whereas Xpert MTB/RIF assay was able to detect nearly 78%, similar to other studies in which Xpert MTB/RIF assay detected 2- to 3-fold more cases than smear microscopy among the culture-positive group (15, 18, 19). Published literature shows a variable sensitivity of Xpert MTB/RIF assay (57–90%) in various samples like gastric aspirate, induced sputum, and nasopharyngeal aspirate (17–21).

There were 305 cases that were symptomatic, had a chest X-ray suggestive of TB, and had either a positive TST or history of close contact with TB case but were culture negative. Xpert MTB/RIF assay was positive in 18% (55/305) of the cases that were negative using the reference standard (liquid culture); 55% of these patients had been classified as PPD.

Of the patients enrolled in the study, 126 (31.9%) children presented with PPD, 204 (51.8%) with PPC, 26 (6.6%) had pleural effusion, while 38 children had no signs of TB and were grouped as “Unlikely TB.” The ratio of PPD to PPC was similar across different age groups. Both liquid culture positivity and Xpert MTB/RIF positivity were significantly higher in the PPD group. This brings out the poor diagnostic yield of laboratory detection methods in the gastric aspirate and induced sputum in children with PPC and pleural effusion.

Xpert MTB/RIF assay diagnosed a considerable number of cases more than the MGIT culture. These cases would have been missed if culture had been used as the only diagnostic modality. Several published reports have shown Xpert positive culture negative cases (4.5–8.5%) (16–19). Xpert MTB/RIF assay is a highly specific test as shown by the published literature (94–99%). Reports of Xpert MTB/RIF assay test results are available earlier in 90 min compared with MGIT culture (10–15 days). This might be useful in cutting down the transmission of the disease due to early initiation of treatment.

The determinants of Xpert MTB/RIF assay and MGIT culture positivity were looked for, and it was found that both were higher in children with PPD. Progressive primary disease is a severe form of illness because of the considerable lung damage that takes place and, hence, is associated with higher bacillary load and a better detection by both methods.

Several studies have reported an independent association of Xpert MTB/RIF assay positivity with age group >5 years, a positive TST, and a history of close contact with a TB case. In the present study, there were three culture-positive rifampicin resistance cases, which were found to be rifampicin resistant by Xpert MTB/RIF as well. Among the culture negative, 55 cases detected by Xpert MTB/RIF assay, three cases were detected to be rifampicin resistant. In one study, of the 48 rifampicin-resistant cases detected by Xpert MTB/RIF, 47 were found to be rifampicin resistant on confirmatory culture DST/LPA (22).

Studies from different parts of the world have shown a sensitivity ranging from 57 to 79% in induced sputum. One study demonstrated Xpert MTB/RIF sensitivity of 90% in sputum samples and 68.8% in gastric aspirate samples (20). In a meta-analysis on Xpert MTB/RIF assay for the diagnosis of pulmonary TB in children published by Detjen et al., pooled sensitivities of Xpert for TB detection were 62% for expectorated or induced sputum, while it was 66% with samples from gastric lavage (23). Bunyasi et al. found sensitivity of Xpert MTB/RIF assay as 26.7% for two induced samples and 22.6% for two gastric lavage samples (24).

In conclusion, 58% of the children in culture-negative TB group had PPC. The cases detected by Xpert MTB/RIF assay in this group had PPD in 55% of the cases. PPC represents disease localized to the lymph nodes with little or no parenchymal involvement and, hence, pauci-bacillary airway samples. Cruz et al. ascribed the disease pathogenesis in PPC to profound inflammatory response to a low bacillary burden (15). The PPD disease, as defined radiologically, has some lung parenchymal activity, and hence, the airway samples may yield positive results. The airway samples may not be the best representative samples for all forms of intra-thoracic disease in children. The possibility of obtaining a more representative sample such as endobronchial biopsy or endoscopic ultrasound-guided sampling of hilar lymph nodes may contribute to the laboratory confirmation of TB. Research studies are investigating improvement in diagnostic yield with samples such as stool, and the latest molecular testing platform, Genexpert Ultra (the authors are part of a multi-centric study by FIND). Nicol et al. (25) found a higher sensitivity of Genexpert Ultra in comparison with Genexpert but less in comparison with culture, in banked induced sputa. Zar et al. (26) have used Genexpert Ultra and recommended the use of combinations of specimens for diagnosis of confirmed pulmonary TB in children. Cochrane review by Kay et al. concluded higher sensitivity in gastric aspirate samples followed by sputum stool, and nasopharyngesl aspirate, while the specificity was >98%. Genexpert Ultra had higher sensitivity but lower specificity than Xpert in data from 299 studies (27).

Limitations of the study include prospective enrollment of consecutive patients with presumptive ITTB; hence, fewer children were enrolled from <5 years age group. The study was set in a high-TB endemicity country and was designed to address the question of improving diagnostic modalities for symptomatic children. The question of diagnosing TB in asymptomatic children could not be built into the present study design. Chest X-ray was done for diagnosis and treatment, although some findings may be missed, as it was not feasible to perform CT scan in all the children.

The study is one of the few from India, designed to assess the diagnostic performance of liquid culture and Xpert MTB/RIF assay in ITTB in children. The current study highlights the correlation between the clinical presentations of TB and yield of sensitive laboratory tests including Xpert MTB/RIF assay. Marais et al. had shown poor microbiological confirmation in lymph node disease, Ghon focus, and pleurisy in children (28). The current study confirms that parenchymal involvement on CXR is an important determinant of increased microbiological yield from respiratory specimen prompting suggestions of alternate samples/newer diagnostic tests for better diagnosis in children with PPC.

## Data Availability Statement

The raw data supporting the conclusions of this article will be made available by the authors, without undue reservation.

## Ethics Statement

The studies involving human participants were reviewed and approved by Institution Ethics Committee, All India Institute of Medical Sciences, New Delhi. Written informed consent to participate in this study was provided by the participants' legal guardian/next of kin.

## Author Contributions

US conceived and designed the study, drafted and revised the article critically for intellectual content, and overall supervised the study. YV was in charge of the patient enrollment, sample collection and transport to the laboratory, and running laboratory tests. RJ handled the patient enrollment, filling the study proforma, obtaining consent, providing patient information sheet (PIS), and patient follow-up. AM also handled the patient enrolment, filling the study proforma, obtaining consent, providing patient information sheet (PIS), patient follow-up, and analysis of data. HG analyzed and interpreted the data and performed the statistical analysis. RL also analyzed and interpreted the data and was in charge of the clinical correlation of results and treatment follow-up. SK was in charge of the selection of the cases and controls, enrollment and treatment follow-up, and supervised the findings of the work. All authors contributed to the article and approved the submitted version.

## Conflict of Interest

The authors declare that the research was conducted in the absence of any commercial or financial relationships that could be construed as a potential conflict of interest.

## Publisher's Note

All claims expressed in this article are solely those of the authors and do not necessarily represent those of their affiliated organizations, or those of the publisher, the editors and the reviewers. Any product that may be evaluated in this article, or claim that may be made by its manufacturer, is not guaranteed or endorsed by the publisher.
