# Supplementary material for: Childhood Intra-Thoracic Tuberculosis Clinical Presentation Determines Yield of Laboratory Diagnostic Assays
Source: Front Pediatr. 2021 Aug 25;9:667726. doi: 10.3389/fped.2021.667726 (PMC8425475; doi:10.3389/fped.2021.667726)
Supplement: Supplementary file 1 [file Data_Sheet_1.docx]

**SOP for Sputum induction:**

1. Sputum induction will be done on the appointed days.
2. It will be done in the pediatric outpatient department of respective hospitals in the morning hours.
3. It will be done by the study nurse.
4. Children will be asked to come at the appointed time after minimum 3 hours of fasting.
5. The oxygen saturation will be preferably measured using a pulse oxymeter at the beginning and end of the procedure.
6. The children will be premedicated with salbutamol (0.15 mg/kg) inhalation by nebulizer or 200 microgram of salbutamol by 2 puffs (salbutamol MDI) along with spacer (mask if required).
7. Then a jet nebuliser attached to oxygen at a flow rate of 5 L per minute will be used to deliver 3-5 mL of 3% sterile saline for 15 minutes.
8. If child is able to expectorate, he/ she will collect sputum after coughing in container directly. If not able to expectorate: chest percussion will be done and Nasopharyngeal secretions will be collected by suctioning with a sterile mucus extractor.
9. Nasopharyngeal suction will be done by introducing an appropriately small sized (no. 7) feeding tube via the nostrils after measuring the halfway distance between tragus and nose.
10. 1 ml of normal saline will be instilled by the feeding tube and then withdrawn using 20 ml syringe or mucus extractor.
11. Specimen will be collected in sterile containers and properly labeled with name, study ID, date and specimen no.1/2, visit no.
12. Entry will be made of the same in the CRF.
13. Specimens will be transported directly to the laboratory for processing by the person responsible.
14. Nebuliser chamber will be sterilized by dipping in 1% acetic acid for 10 minutes.
15. Any adverse events during this procedure will be duly recorded.
16. Same procedure will be repeated on the next day at an appointed time.

**SOP 6**

**Gastric aspiration**

1. Position the child on his or her back or side. The assistant should help to hold the child.
2. Measure the distance between the nose and stomach, to estimate distance that will be required to insert the tube into the stomach.
3. Attach a syringe to the nasogastric tube.
4. Gently insert the nasogastric tube through the nose and advance it into the stomach.
5. Withdraw (aspirate) gastric contents (2-5ml) using the syringe attached to the nasogastric tube.
6. To check that the position of the tube is correct, push some air (e.g. 3-5 ml) from the syringe into the stomach and listen with a stethoscope over the stomach.
7. If no fluid is aspirated, insert 5-10 ml sterile water or normal saline and attempt to aspirate again.

- If still unsuccessful, attempt this again (even if the nasogastric tube is in an incorrect position and water or normal saline is inserted into the airways, the risk of adverse events is still very small).
- Do not repeat more than three times.

1. Withdraw the gastric contents (ideally at least 5-10 ml).
2. Transfer gastric fluid from the syringe into a sterile container (sputum collection cup)
